# 34.1 DO ALL INDIVIDUALS WITH A FEP PASS THROUGH AN EARLIER CHR-P STATE? IMPLICATIONS FOR CLINICAL STAGING, EARLY DETECTION AND PHASE-SPECIFIC INTERVENTIONS

**DOI:** 10.1093/schbul/sby014.142

**Published:** 2018-04-01

**Authors:** Jai Shah, Rachel Rosengard, Sarah McIlwaine, Sally Mustafa, Srividya Iyer, Martin Lepage, Ridha Joober, Ashok Malla

**Affiliations:** McGill University

## Abstract

**Background:**

The CHR-P syndrome has attracted much attention as a potentially important stage for early intervention aimed at preventing or delaying the onset of psychosis. Knowledge regarding the transition from CHR-P to FEP has been widely described and disseminated, but a major (untested) assumption permeates this literature: that most or all patients with a FEP actually experienced an earlier CHR-P state. Examining this assumption will provide crucial information regarding the potential utility of public mental health efforts such as early case identification and prevention directed at the CHR-P stage.

**Methods:**

Semistructured interviews of 351 patients and families with the Circumstances of Onset and Relapse Schedule were supplemented by chart reviews in a catchment area–based sample of FEP patients in Montréal, Canada. Retrospective information was extracted regarding baseline sociodemographic variables, psychiatric and behavioral changes, and help-seeking behavior up to the point of intake in the FEP service. Experts (N=30) working in FEP and CHR settings identified which of 27 early signs and symptoms in the Topography of Psychotic Episode instrument constituted sub-threshold psychotic symptoms if they appeared prior to a syndromal-level psychotic episode. Individuals were then followed within the FEP service for up to 2 years in order to record a range of symptomatic (positive and negative symptoms, depression and anxiety) and functional (global functioning, social and occupational functioning) outcomes.

**Results:**

While most clients (between 50–68%) experienced at least one early sub-threshold psychotic symptom prior to their FEP, a substantial minority recalled no CHR-P symptoms en route to psychosis. At entry to FEP services, there were no differences in sociodemographic, cognitive, or functional variables between youth who had experienced a CHR-P state versus those who had not. Youth with a CHR-P profile had significantly longer durations between psychosis onset and making the decision to seek help (median 7.7 weeks versus 3.7 weeks), as well as the total length of the prodrome leading up to psychosis (median 36.4 weeks versus 15.0 weeks). These subgroups also differed in key symptomatic and functional outcomes, with those who passed through CHR-P states en route to FEP having significantly higher depressive and anxiety symptoms at baseline, more positive and negative psychotic symptoms at 1 year, and lower functioning for at least 1 year after the initiation of FEP treatment.

**Discussion:**

A substantial minority of FEP cases did not recall a CHR-P state, suggesting that a wide range of psychopathology precedes FEP. Nonetheless, our estimates indicate that over 50% of FEP cases could still be prevented through optimal interventions targeting the CHR-P phase. This adds a novel component to previous arguments regarding the feasibility and relevance of the CHR-P construct for FEP, and underscores the importance of early case identification for this vulnerable population. Implications of these findings for contemporary clinical staging models, prevention and intervention efforts will be discussed.

